# Pharmacogenomics of dolutegravir: A scoping review of evidence, gaps and clinical implications

**DOI:** 10.1002/bcp.70721

**Published:** 2026-07-28

**Authors:** Ronald Kiguba, Mishael Ssesanga, Munir Pirmohamed

**Affiliations:** ^1^ Department of Pharmacology and Therapeutics, College of Health Sciences Makerere University Kampala Uganda; ^2^ Centre for Drug Safety Science and Wolfson Centre for Personalised Medicine, Institute of Systems, Molecular and Integrative Biology University of Liverpool Liverpool UK

**Keywords:** adverse drug reactions, clinical pharmacology, dolutegravir, pharmacodynamics, pharmacogenomics, pharmacokinetics

## Abstract

Dolutegravir underpins modern first‐ and second‐line HIV treatment regimens; however, interindividual variability in its disposition and tolerability presents challenges for optimal use. This scoping review mapped current evidence on the pharmacogenomics of dolutegravir, focusing on pharmacokinetics and pharmacodynamics, and methodological limitations of existing studies. Reduced‐function *UGT1A1* alleles (*6, *28, *37) emerged as the most consistent determinants of increased dolutegravir exposure across African, European and Asian populations, reinforcing *UGT1A1* as the predominant genetic pathway for dolutegravir clearance. Transporter polymorphisms, particularly in *ABCG2*, showed variable associations with dolutegravir concentrations, with differing results in children compared with adults. Genome‐wide association studies in African populations have identified novel candidate loci (e.g., *CAMKMT* and *MIR99AHG*) requiring replication. Evidence linking genetic variation to clinical outcomes remains weak: Associations with neuropsychiatric adverse events (notably in *UGT1A1* and *SLC22A2*), weight gain (*ABCG2*, *MC4R* and *TMEM163*) and viral suppression were inconsistent and underpowered. Suggestive gene–gene and gene–drug interactions merit further investigation. The current evidence base is constrained by small sample sizes, heterogeneous study designs, overlapping datasets, non‐adjustment for multiple testing, limited ancestral diversity and inconsistent phenotyping. Overall, *UGT1A1* variation is the only reproducible pharmacogenomic signal of clinical relevance; however, its effect size does not currently justify routine genotype‐guided dosing. In the future, priority should be given to well‐powered, multi‐ancestry, harmonised studies with functional validation to clarify the role of pharmacogenomics in tailoring dolutegravir‐based therapy.

## 1 INTRODUCTION

Dolutegravir is a widely used second generation integrase strand transfer inhibitor (INSTI) recommended as the anchor drug of first‐line and second‐line combination antiretroviral therapy (cART) in many countries, particularly in low‐ and middle‐income countries (LMIC) where the HIV burden is highest. It is highly potent, with a favourable safety profile, and has a high genetic barrier to drug resistance.^1^ Additionally, dolutegravir is rapidly absorbed and has a long half‐life, enabling once‐daily dosing without the need for pharmacokinetic boosting.^2^ However, individual responses to dolutegravir vary, with some individuals experiencing reduced efficacy or tolerance. This interindividual variability in response to dolutegravir may be influenced by both genetic factors, such as polymorphisms in drug‐metabolising enzymes and transporters, and non‐genetic factors, such as age, sex, ethnicity, comorbidities and co‐medications.^3^

Pharmacogenomics—the study of how genetic variation influences individual responses to drugs—has the potential to improve our understanding of the variability in dolutegravir pharmacokinetics and pharmacodynamics.^3^, ^4^ Nearly 80% of dolutegravir is metabolised in the liver via glucuronidation, primarily by the uridine diphosphate glucuronosyltransferase 1A1 (UGT1A1) enzyme, encoded by the *UGT1A1* gene, with minor contributions from UGT1A3, UGT1A8 and UGT1A9.^5^, ^6^ The *UGT1A1* genetic variants UGT1A1*1 is associated with normal UGT1A1 activity, while *6, *28 and *37 are associated with reduced UGT1A1 activity and UGT1A1*36 with increased UGT1A1 activity.^7^, ^8^ UGT1A1*60 is a promoter variant found in the phenobarbital‐responsive enhancer module and would be expected to decrease transcriptional activity. However, it is in strong linkage disequilibrium with UGT1A1*28,^9^ and therefore, its individual impact is unclear.

The combination of two *UGT1A1* alleles determines whether an individual has a poor metaboliser, intermediate metaboliser or extensive metaboliser phenotype. The Clinical Pharmacogenetics Implementation Consortium (CPIC) uses the following categories for UGT1A1 genotype‐predicted phenotypic function:

- extensive metabolisers (*1/*1, *1/*36, *36/*36),
- intermediate metabolisers (*1/*28, *1/*37, *36/*28, *36/*37, *1/*6) and
- poor metabolisers (*28/*28, *28/*37, *37/*37, *6/*6).^7^

About 10% to 15% of dolutegravir is metabolised by oxidation via cytochrome P450 3A (CYP3A), mainly CYP3A4.^5^, ^10^ Other minor metabolic pathways of dolutegravir include CYP1A1 which is primarily expressed in the respiratory system and CYP1B1 in several extrahepatic tissues.^11^, ^12^, ^13^ Dolutegravir is also a substrate of the efflux transporter proteins P‐glycoprotein (P‐gp), encoded by *ABCB1*, and breast cancer resistance protein (BCRP), encoded by *ABCG2*.^14^ Furthermore, nuclear receptors, such as *NR1I2* (pregnane X receptor [PXR]) regulate the expression of key drug‐metabolising enzymes and transporters, including *UGT1A1*, *CYP3A4* and *ABCB1*.^15^

In terms of dolutegravir tolerability, premarket studies demonstrated a favourable safety profile, with neuropsychiatric adverse events leading to discontinuation in <1% of cases. However, in real‐life settings, discontinuation rates ranging from 1.4% to 7.2% have been associated with neuropsychiatric adverse events.^16^, ^17^, ^18^, ^19^, ^20^, ^21^, ^22^, ^23^, ^24^ To date, the available evidence remains insufficient to support the routine implementation of pharmacogenomic testing.^3^

Several pharmacogenomic studies have shown inconsistent findings regarding the association between certain genetic variants and treatment response or toxicity to dolutegravir‐based cART, which limits generalisability. These inconsistent findings could result from methodological challenges in current pharmacogenomic studies in terms of study design, sample size, genotyping strategies and phenotyping strategies, among others. Phenotypic approaches include markers of drug concentration (pharmacokinetic measures, e.g., *C*_max_, *C*_min_, *C*_tau_, drug clearance, AUC and half‐life) and clinical outcomes (pharmacodynamic measures, e.g., viral suppression and adverse events). A scoping review was therefore selected as the most appropriate design to map the existing evidence across heterogeneous study designs and to identify research gaps, rather than to provide a pooled estimate of effect sizes. In this review, our aim is to summarise the current knowledge of the pharmacogenomics of dolutegravir, with a focus on pharmacokinetics and pharmacodynamics, and to highlight the strengths and limitations of existing studies.

## 2 METHODS

### 2.1 Specific objectives

1. To summarise the current knowledge on the pharmacogenomics of dolutegravir focusing on its pharmacokinetics and pharmacodynamics and any interplay between genetic and non‐genetic factors.
2. To evaluate the strengths and limitations of existing studies on the pharmacogenomics of dolutegravir with emphasis on study design, sample size, genotyping strategy and phenotyping strategy.

### 2.2 Study design

This review followed the Joanna Briggs Institute (JBI) guidelines for scoping reviews^25^ and Preferred Reporting Items for Systematic Reviews and Meta‐Analyses Extension for Scoping Reviews (PRISMA‐ScR).^26^ The protocol used the JBI scoping review template and was registered with Open Science Framework (https://doi.org/10.17605/OSF.IO/WHNJU).

### 2.3 Eligibility criteria

The inclusion criteria for selecting publications, with no language restrictions, were peer‐reviewed articles and grey literature on dolutegravir pharmacogenomics from primary studies conducted in human populations. Literature review articles were excluded as part of the predetermined criteria to minimise the duplication of primary research evidence, and other publications were omitted if they did not meet the specific objectives of the scoping review.

### 2.4 Search strategy

We designed a comprehensive search strategy to identify all relevant publications related to the research questions. Peer‐reviewed articles were searched in several databases, including PubMed, EBSCOhost, Web of Science, Wiley Online Library and Google Scholar, from inception to 18 August 2025. We also screened the reference lists of included publications. The search strings for peer‐reviewed articles are provided in Data S1. We identified the grey literature by searching ProQuest Dissertations & Theses, World Health Organization Institutional Repository for Information Sharing, ClinicalTrials.gov, pharmacogenomics databases^27^, ^28^, ^29^, ^30^ and conference proceedings. Inclusion criteria for grey literature matched those for peer‐reviewed articles, except that preprints, reports and abstracts with sufficient methodological detail were eligible.

### 2.5 Study selection

Two reviewers (R.K. and M.S.) independently screened all titles and abstracts of the identified peer‐reviewed articles for eligibility. The full texts of potentially eligible peer‐reviewed articles were retrieved and independently assessed for inclusion by the two reviewers. The grey literature was similarly reviewed. Disagreements were resolved by consensus or in consultation with a third reviewer (M.P.).

### 2.6 Data extraction

The data were extracted by R.K. and M.S. using a tool created by the reviewers (Data S2). This tool was designed to capture various parameters, including the author, journal, title of study, objectives, study design, sample size, country, year of publication, phenotyping approach, genotyping approach and ancestry. The data were synthesised narratively following the JBI recommendations.^25^, ^26^ The extracted data were reviewed by R.K. and M.S. and revised accordingly.

### 2.7 Data analysis and presentation

Extracted data were summarised following the scoping review objectives. Risk of bias or quality assessment were not conducted on the included publications in accordance with the JBI guidelines which do not require scoping reviews to appraise the risk of bias for a cumulative body of evidence.^26^ We focused on mapping the existing evidence and identifying knowledge gaps rather than estimating effect sizes.

### 2.8 Patient and public involvement

Patients and the public did not participate in the design, conduct and reporting of this research.

## 3 RESULTS

### 3.1 Overview of publications

The selection of publications is shown in the PRISMA‐ScR flow diagram (Figure 1). We identified 21 publications (19 peer‐reviewed and two grey literature) (Tables 1 and 2).

**FIGURE 1:**
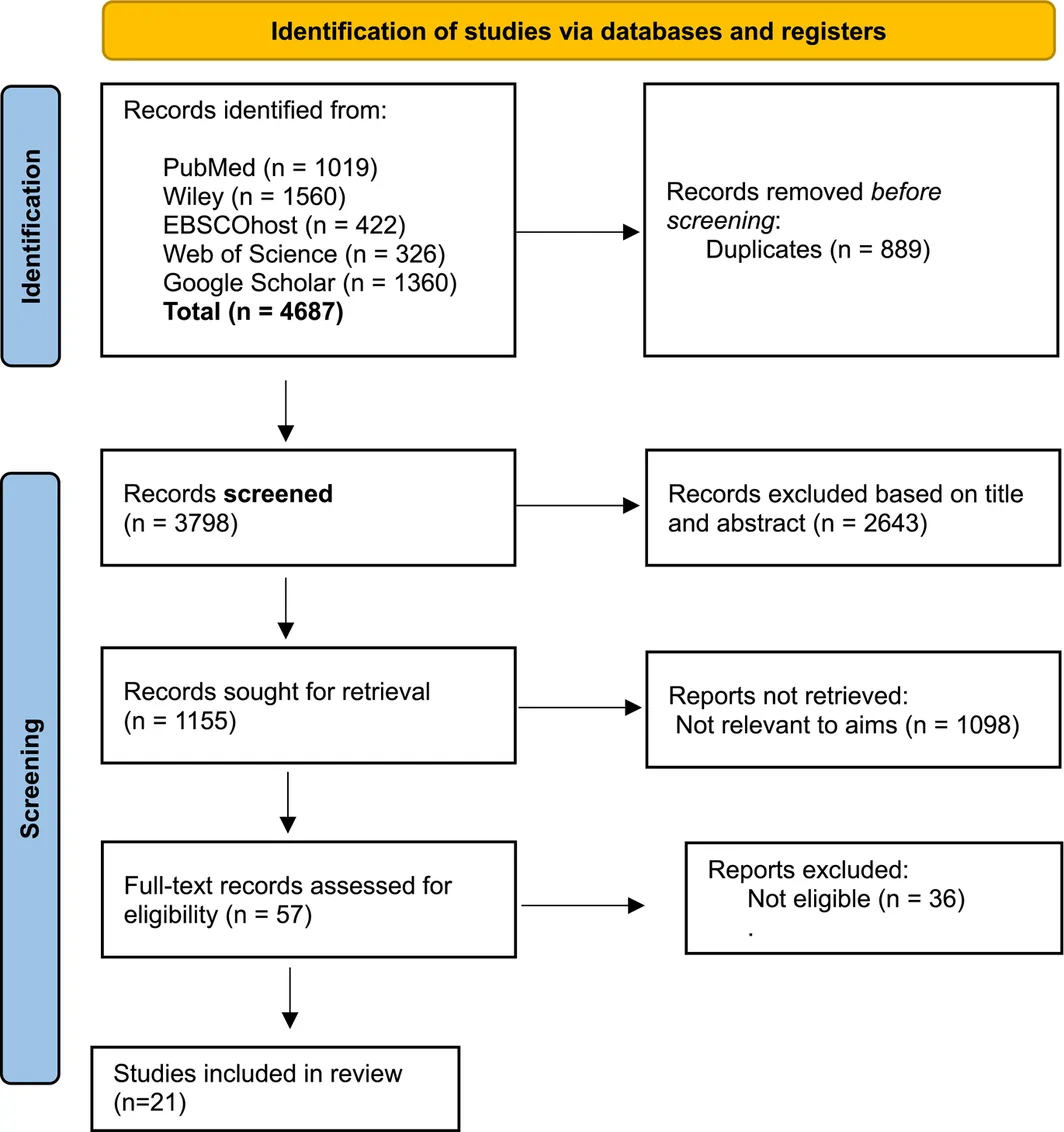
PRISMA‐ScR flow diagram.

**TABLE 1:** List of included peer‐reviewed dolutegravir‐related pharmacogenomics publications.

First author (year)	Journal	Title	Objectives	Study design	Sample size	Country	Phenotyping approach		Genotyping approach		Ancestry			
							PK	PD	Targeted	GWAS	African	European	Asian	Unknown/others
Haas D. W. (2021)	Clin Infect Dis	Implications of efavirenz pharmacogenetics when switching from efavirenz‐ to dolutegravir‐containing antiretroviral regimens	To identify the implications of switching from efavirenz to dolutegravir regimens	Clinical trial (Phase I)	9 of 12 HIV negative	United States	✓		✓			✓		✓
Chen S. (2014)	Pharmacogenomics	Evaluation of the effect of UGT1A1 polymorphisms on dolutegravir pharmacokinetics	To evaluate potential pharmacogenetic effects of UGT1A1 polymorphisms on the pharmacokinetics of dolutegravir	Pooled analysis of nine clinical trials (phases I and II)	89	United States	✓	✓	✓		✓	✓	✓	
Cindi Z. (2022)	J Infect Dis	Pharmacogenetics of dolutegravir plasma exposure among southern Africans with human immunodeficiency virus	To characterise genetic association with DTG exposure among ART‐naïve African participants	Randomised clinical trial (phase III)	284 of 1053 in RCT	South Africa	✓	✓	✓	✓	✓			
Elliot E. R. (2020)	J Antimicrob Chemother	Genetic influence of ABCG2, UGT1A1, NR1I2 on dolutegravir plasma pharmacogenetics	To investigate the role of common UGT1A1, ABCG2, CYP3A and NR1I2 SNPs on plasma dolutegravir concentrations	Pooled analysis of three phase I and one phase III trial	93	United Kingdom	✓	✓	✓		✓	✓	✓	✓
Griesel R. (2022)	J Antimicrob Chemother	Pharmacokinetic and pharmacogenetic associations with dolutegravir neuropsychiatric adverse events in an African population	To determine in an African population whether a concentration–response relationship exists between dolutegravir and treatment neuropsychiatric events	Randomised clinical trial (phase III)	301	South Africa		✓	✓	✓	✓			
Borghetti A. (2019)	J Antimicrob Chemother	SLC22A2 variants and dolutegravir levels correlate with psychiatric symptoms in persons with HIV	To investigate the potential association between SLC22A2 gene variants and dolutegravir concentration with neuropsychiatric symptoms	Cross‐sectional	203	Italy		✓	✓			✓		✓
Cusato J. (2022)	Metabolites	Dolutegravir discontinuation for neuropsychiatric symptoms in people living with HIV and their outcomes after treatment change: a pharmacogenetic study	To evaluate the influence of the SLC22A2 808 C > A polymorphism on the incidence of dolutegravir discontinuation in people living with HIV	Prospective observational cohort	627	Italy		✓	✓			✓		✓
Tsuchiya K. (2017)	Pharmacogenet Genomics	High plasma concentrations of dolutegravir in patients with ABCG2 genetic variants	To determine SNPs of ABCB1 and ABCG2 genes and plasma DTG concentrations	Cross‐sectional	42	Japan	✓		✓				✓	
Mpofu R. (2024)	Br J Clin Pharmacol	Determinants of early change in serum creatinine after initiation of dolutegravir‐based antiretroviral therapy in South Africa	To assess clinical, pharmacokinetic and genetic factors associated with change in serum creatinine after initiation of dolutegravir‐based antiretroviral therapy	Randomised clinical trial (phase III)	315	South Africa		✓	✓	✓	✓			
Yagura H. (2017)	BMC Infect Dis	Impact of UGT1A1 gene polymorphisms on plasma dolutegravir trough concentrations and neuropsychiatric adverse events in Japanese individuals infected with HIV‐1	To examine the relationship between the plasma trough concentration of dolutegravir and UGT1A1 gene polymorphisms (*6/*28)	Cross‐sectional	107	Japan	✓	✓	✓				✓	
Spector S. A. (2024)	J Acquir Immune Defic Syndr	Impact of genetic variants in ABCG2, NR1I2, and UGT1A1 on the pharmacokinetics of dolutegravir in children	To examine the impact of genetic variants in ABCG2 421C > A (rs2231142), NR1I2 63396 C > T (rs2472677) and UGT1A1 (rs5839491) on dolutegravir PK	Cross‐sectional	59	United States	✓		✓		✓	✓	✓	
Cindi Z. (2021)	J Acquir Immune Defic Syndr	Genetic associations with weight gain among South Africans who initiated dolutegravir and tenofovir‐containing regimens	To characterise associations between human genetic polymorphisms and the magnitude of weight gain among ART‐naïve individuals randomised to initiate dolutegravir‐containing regimens	Clinical trial (phase III)	314	South Africa		✓	✓	✓	✓			
Griesel R. (2021)	Clin Infect Dis	CYP2B6 genotype and weight gain differences between dolutegravir and efavirenz	To determine the effect of CYP2B6 metaboliser genotype on weight gain in a subset of participants randomised to efavirenz and dolutegravir in the ADVANCE study	Clinical trial (phase III)	510	South Africa		✓	✓	✓	✓			
Leonard M. A. (2021)	Clin Infect Dis	Efavirenz pharmacogenetics and weight gain following switch to integrase inhibitor‐containing regimens	To characterise associations between CYP2B6 and UGT1A1 genotype and weight gain at Week 48 in an observational cohort of ART‐experienced patients who switched from efavirenz‐containing to INSTI‐containing regimens during clinical care. To characterise associations between CYP2B6 genotype and weight gain at Week 48 in a clinical trial cohort of ART‐naïve participants randomised to efavirenz‐containing regimens in AIDS Clinical Trials Group (ACTG) studies but did not receive INSTIs	Retrospective observational cohort and retrospective clinical trial (phase III)	523 (61—observ. cohort; 462—clinical trials cohort)	United States		✓		✓	✓	✓	✓	✓
Wu K. (2024)	Pharmacogenet Genomics	Pharmacogenetics of weight gain following switch from efavirenz‐ to integrase inhibitor‐containing regimens	To characterise associations between CYP2B6 genotype and weight gain after ART switch among ACTG A5001 and A5322 participants	Retrospective observational cohort	159 (67 on dolutegravir)	United States		✓		✓	✓	✓	✓	✓
Griesel R. (2024)	J Acquir Immune Defic Syndr	Pharmacokinetics of single‐dose versus double‐dose dolutegravir after switching from a failing efavirenz‐based regimen	To determine if dolutegravir exposure is reduced after switching from efavirenz	Clinical trial (phase II)	35	South Africa	✓		✓		✓			
Kengo A. (2023)	Antimicrob Agents Chemother	Dolutegravir pharmacokinetics in Ugandan patients with TB and HIV receiving standard‐ versus high‐dose rifampicin	To predict target attainment in a large cohort of in silico individuals using different dosing strategies	Phase II clinical trial	44	Uganda	✓		✓		✓			
Covington N. (2025)	Pharmacogenet Genomics	Pharmacogenetics of plasma dolutegravir exposure during 1‐month rifapentine/isoniazid treatment of latent tuberculosis	To characterise the impact on dolutegravir *C*_trough_ of UGT1A1, AADAC and NAT2 polymorphisms that affect dolutegravir, rifapentine and isoniazid, respectively	Cross‐sectional	30	United States	✓		✓		✓	✓	✓	
Murrell D. E. (2019)	Clin Drug Investig	HIV integrase inhibitor pharmacogenetics: an exploratory study	To examine the role of SNPs on drug concentrations, selected clinical outcomes and adverse events	Cross‐sectional	88	United States	✓	✓	✓			✓		✓
De Greef J. (2025)	J Infect Dis	Dolutegravir and risk of neuropsychiatric adverse events: a pharmacogenetic study	To determine the association between NPAE‐related DTG discontinuation and selected pharmacogenetic polymorphisms	Prospective cohort	137	Belgium	✓	✓	✓		✓	✓		

**TABLE 2:** List of included grey literature on dolutegravir‐related pharmacogenomics.

First author (year)	Journal	Title	Objectives	Study design	Sample size	Country	Phenotyping approach		Genotyping approach		Ancestry			
							PK	PD	Targeted	GWAS	African	European	Asian	Unknown/others
Ridolfi F. (2024)	Res Sq	Pharmacogenomic associations with HIV‐1 virologic suppression in TB/HIV patients	To evaluate the associations between genetic variants of antiretroviral therapy (ART) and HIV‐1 outcomes among TB/HIV patients	Prospective observational cohort	194	Brazil		✓	✓		✓	✓	✓	✓
Hulgan T. (2025)	Open Forum Infect Dis	Melanocortin‐4 receptor gene variants and weight change following switch to integrase inhibitor‐containing antiretroviral therapy	To examine associations between MC4R gene variants and weight gain following switch to INSTI‐containing ART among PWH in ACTG observational cohort studies A5001 and A5322	Retrospective observational cohort	530	United States		✓		✓	✓	✓		✓

### 3.2 Genetic variants associated with dolutegravir metabolism

#### 3.2.1 UGT1A1

A meta‐analysis of nine clinical trials (eight phase I and one phase II) including 89 participants (52% European‐Americans, 42% African‐Americans) suggested that Individuals with reduced UGT1A1 activity had a 32% lower CL/F, a 46% higher AUC_0−*t*_ and a 32% higher *C*_max_ compared with individuals with normal activity.^31^ Specifically, UGT1A1*28/*28, *28/*37, *1/*6, *1/*28, *1/*37, *28/*36 and *36/*37 were associated with decreased dolutegravir clearance as compared with UGT1A1*1/*1 and *1/*36. Individuals with low (*28/*28, *28/*37) and reduced enzyme activity (*1/*6, *1/*28, *1/*37, *28/*36, *36/*37) polymorphisms had decreased oral dolutegravir clearance (CL/F; 23% decrease), increased AUC_0−*t*_ (31%) and higher maximum plasma concentrations (*C*_max_; 22%) compared with those with normal activity genotypes (*1/*1, *1/*36).^31^ Furthermore, a pooled analysis of four clinical trials involving 93 participants, of whom 57 were HIV‐infected and 36 healthy volunteers (75% Caucasian, 17% African, 3% Asian, 1% mixed race, 3% other), found associations between plasma dolutegravir exposure and genetic variants in *UGT1A1*.^27^, ^32^ Combined UGT1A1*28 and UGT1A1*6 diplotypes yielding genotypic scores ≥3 were associated with a 36% increase in dolutegravir AUC_0–24_ (*β* = .041, *P* = .023) and a 44% increase in *C*_min_ (*β* = .042, *P* = .009).^32^ The study further observed that plasma dolutegravir AUC_0–24h_ was 27% higher in subjects with the UGT1A1 poor metaboliser phenotype (*28/*28, *28/*37 or *37/*37) than in those with the extensive or intermediate metaboliser phenotypes (*P* = .020).^32^

Among 59 children in the United States (median age 4.6 years, interquartile range 1.6–10.0) who received a 5 mg dispersible dolutegravir tablet, intermediate metabolisers exhibited lower dolutegravir concentrations than normal metabolisers, with a significantly decreased adjusted AUC_24_ (*P* = .021). Furthermore, poor metabolisers (*n* = 5) showed non‐significant trends towards higher exposure including modest increases in log_10_ *C*_max_ (*P* = .37) and log_10_ *C*_min_ (*P* = .25) and reduced clearance (*P* = .16), compared with the combined intermediate and normal groups.^33^

In Black South Africans, the UGT1A1*80 T‐allele, a surrogate marker for *28 and *37, was associated with increased dolutegravir concentration (AUC). In particular, the UGT1A1*80 C/T and T/T genotypes were associated with 11% and 26% decreases in dolutegravir clearance, respectively, and therefore higher plasma dolutegravir exposure compared with C/C individuals.^34^ Another *UGT1A1* locus polymorphism, rs28899168, was associated with increased UGT1A1 expression or enzyme activity independent of UGT1A1*80. Adjustment for UGT1A1*80 in the population pharmacokinetic model showed that rs28899168 was independently associated with lower plasma dolutegravir concentration (AUC); however, this association was wiped out after adjustment for multiple testing. The association between rs28899168 in *UGT1A1* and dolutegravir pharmacokinetics has not previously been documented.^34^ In an open‐label, multicentre clinical trial of 30 people living with HIV, UGT1A1*80 was associated with dolutegravir trough concentration at Days 0 and 28. Day 28 dolutegravir *C*_trough_ was higher with T/T and C/T than with C/C genotypes.^35^

#### 3.2.2 CYP3A

Pooled analysis of four clinical trials involving 93 participants in United Kingdom did not find any associations between dolutegravir *C*_max_ and CYP3A4*22 or CYP3A5*3.^32^ Similarly, a pharmacogenetic analysis of 284 participants in a South African clinical trial found no associations between plasma dolutegravir exposure and polymorphisms in *CYP3A4* and *CYP3A5*.^34^ Furthermore, Chen et al.^31^ demonstrated that dolutegravir‐pharmacokinetic parameters were similar across CYP3A4 and CYP3A5 functional groups (low, reduced and normal metabolisers). No statistically significant differences were observed in dolutegravir exposure (CL/F, AUC and *C*_max_) among individuals with low activity markers for CYP3A4 and CYP3A5.^31^

### 3.3 Genetic variants associated with dolutegravir transport

#### 3.3.1 ABCB1 and ABCG2

A Japanese study of 42 participants found an association between the rs2231142 variant in *ABCG2* and higher dolutegravir *C*_max_ (*P* = .0005), while no significant associations were observed with *ABCB1* variants (rs1128503, rs1045642, rs2032582 or rs3842).^36^ Similarly, pooled analysis of four clinical trials with 93 participants (75% Caucasian, 17% African) found that the homozygous variant in *ABCG2* (rs2231142) was associated with a 28% increase in dolutegravir *C*_max_ (*P* = .047). However, no associations were detected between dolutegravir *C*_max_ and another *ABCG2* variant (rs2231137).^32^ By contrast, a South African study of 284 individuals found no association between plasma dolutegravir exposure (AUC) and polymorphisms in *ABCB1* and *ABCG2*.^34^

A US study involving 59 children reported a significant association between having ≥1 minor allele in *ABCG2* at rs2231142 and lower adjusted mean dolutegravir *C*_max_ difference (*P* = .003). However, no significant association was observed between having ≥1 minor *ABCG2* allele(s) and the adjusted mean dolutegravir AUC difference (*P* = .07).^33^

### 3.4 Genetic variants in nuclear receptors regulating the metabolism and transport of dolutegravir

#### 3.4.1 NR1I2

The homozygous variant in *NR1I2*, rs2472677, was associated with a 28% increase in dolutegravir *C*_max_ (*P* = .033). However, no associations were detected between dolutegravir *C*_max_ and *NR1I2* rs1523130.^32^ Cindi et al. found no significant association between plasma dolutegravir exposure (AUC) and these two variants in *NR1I2* (rs2472677 and rs1523130).^34^ Chen et al. also found no significant difference between dolutegravir exposure (CL/F, AUC and *C*_max_) and the *NR1I2* variants.^31^

### 3.5 Other genetic variants associated with dolutegravir pharmacokinetics

In a genome‐wide association study among Black South Africans, variants in *CAMKMT* (rs343942; *P* = 2.4 × 10^−7^) and *MIR99AHG* (rs9980715; *P* = 6.6 × 10^−7^) were associated with dolutegravir pharmacokinetics (AUC).^34^ Murrell et al.^37^ reported that the *CYP2D6* variants, rs1065852, rs3892097 and rs7294 in *VKORC1* were associated with increased dolutegravir concentration, while *SLC01B1* rs4149056 and *CYP2B6* rs8192709 were associated with decreased dolutegravir concentration. The two *CYP2D6* SNPs, rs1065852 and rs3892097, were in linkage disequilibrium (*r* ^2^ = .850). However, the exploratory study by Murrell et al. did not control for multiple testing, and these results have not been reported elsewhere.^37^

### 3.6 Genetic variants associated with dolutegravir‐related adverse events

#### 3.6.1 Neuropsychiatric adverse events

UGT1A1*6 and UGT1A1*28 were associated with a higher risk of neuropsychiatric symptoms.^38^ Also, *SLC22A2* variants alone (coding for organic cation transporter 2 [OCT‐2] which is inhibited by dolutegravir) and in combination with pre‐existing psychiatric comorbidities were associated with dolutegravir discontinuation due to neuropsychiatric symptoms.^39^ In a cross‐sectional study of 203 Italian HIV‐positive individuals, Borghetti et al. found no association between the *SLC22A2* rs316019 variant and plasma dolutegravir *C*_trough_. Similarly, the *SLC22A2* c.808C > G (rs316019) variant was not associated with an abnormal General Symptom Index (GSI) (*P* = .072) but with anxiety (*P* = .044), hostility (*P* = .012) and moderate to severe headache (*P* = .037). Furthermore, higher plasma dolutegravir *C*_trough_ was associated with hostility (*P* = .007) and psychoticism (*P* = .008).^40^ However, a follow‐up study by the same group^39^ in a larger cohort did not confirm a direct association between rs316019 in *SLC22A2* and dolutegravir discontinuation for neuropsychiatric symptoms. Instead, pre‐existing psychiatric comorbidities were the primary predictor of discontinuation, with a complex interaction: *SLC22A2* CA/AA genotypes showed opposite effects depending on comorbidity status.^39^ Additionally, De Greef et al. found no association between key variants in *UGT1A1* or *SLC22A2* (rs316019) and dolutegravir discontinuation due to neuropsychiatric adverse events. Only *NR1I2* c.‐22‐7659C > T was independently associated with reduced risk of neuropsychiatric adverse event‐related dolutegravir discontinuation for T‐allele carriers (OR 0.36, *P* = .02).^41^ Moreover, Griesel et al. observed that rs316019 in the *SLC22A2* gene was not associated with sleep quality or mental health screening scores suggesting that the impact of these polymorphisms could vary depending on the specific central nervous system outcome measured.^42^

Regarding *UGT1A1*, UGT1A1*80 was not associated with sleep quality or mental health screening scores.^42^ However, Yagura et al. observed an association between UGT1A1*6 or UGT1A1*28 and a higher risk of neuropsychiatric symptoms.^38^

#### 3.6.2 Weight gain

In candidate‐gene analyses of 314 Black South Africans, Cindi et al. observed a potential association between *ABCG2* (rs4148149, rs13137622) and dolutegravir‐related percentage weight gain from baseline to Week 48. However, these associations were not significant after correcting for multiple testing.^34^ In genome‐wide association studies, however, *TMEM163* rs7590091 was associated with percentage weight gain from baseline to Week 48 after correcting for multiple testing.^34^

Hulgan et al. evaluated the relationship between melanocortin‐4 receptor ( *MC4R* ) variants and weight gain following a switch to INSTI‐based ART in 530 HIV‐positive individuals. Among the participants, 21% received dolutegravir, 9% received tenofovir alafenamide and 73% switched from protease inhibitors.^43^ Of the 20 *MC4R* variants evaluated, only three were associated with weight gain following INSTI use. The variant rs11873305 was associated with weight gain among all participants (*P* = .01), particularly in males (*P* = .004) and White participants (*P* = .02), while rs56758444 showed the strongest association overall (*P* = .001). However, these relationships did not withstand adjustment for multiple testing.^43^

#### 3.6.3 Changes in serum creatinine

The use of dolutegravir can lead to an increase in serum creatinine because the drug interacts with the same transporters that are responsible for the transport of creatinine, in particular OCT2 (*SLC22A2*) and MATE‐1 (*SLC47A1*).^44^ Mpofu et al. screened 39 genetic variants, of which 36 were deemed evaluable in 315 South Africans. Twelve of the variants were in *UGT1A1*, 15 in *SLC22A2* and nine in *SLC47A1*. None of the 36 evaluated variants were associated with a change in serum creatinine after correcting for multiple testing.^44^ This change in creatinine can be regarded as a biochemical adverse event but has the potential to be misinterpreted as renal toxicity.

### 3.7 Genetic variants associated with dolutegravir‐related viral suppression

Ridolfi et al. investigated the association between UGT1A1*80 and viral suppression (<1000 HIV‐1 RNA viral copies/mL) in 100 HIV‐positive individuals co‐treated with antituberculous therapy and INSTI‐based antiretroviral therapy. Of the 100 individuals, 12 received dolutegravir, while the rest received raltegravir. No association was found between UGT1A1*80 and viral suppression when INSTIs were analysed together.^45^ Furthermore, Spector et al. did not detect any statistically significant associations between *ABCG2* or *UGT1A1* and viral suppression in 53 children (<400 HIV‐1 RNA viral copies/mL).^33^

### 3.8 Interplay between genetic and non‐genetic factors

#### 3.8.1 Gene–gene interactions

Elliot et al. identified stronger pharmacogenetic associations between plasma dolutegravir exposure (*C*_max_, AUC_0–24h_) and the co‐occurrence of genetic variants in *UGT1A1*, *ABCG2* and *NR1I2*. A combination of UGT1A1*28 and *NR1I2* rs2472677 homozygosity was associated with a 79% increase in plasma dolutegravir AUC_0–24h_ (*P* = .001) *vs*. a 27% increase in AUC_0–24h_ when UGT1A1*28 (*P* = .020) and *NR1I2* rs2472677 (*P* = .033) were analysed individually. A combination of *ABCG2* rs2231142 and *NR1I2* rs2472677 was associated with a 43% increase in dolutegravir *C*_max_ (*P* = .002) *vs*. a 28% increase in *C*_max_ when *ABCG2* rs2231142 (*P* = .047) and *NR1I2* rs2472677 (*P* = .033) were analysed individually.^32^ The co‐occurrence of UGT1A1*28 poor metaboliser (*28/*28, *28/37*, 37*/37*) and UGT1A1*6 intermediate metaboliser genotypes was associated with a 43% increase in plasma dolutegravir AUC_0–24h_ (*P* = .023) *vs*. a 27% increase in AUC_0–24h_ when the UGT1A1*28 poor metaboliser status was analysed individually (*P* = .020).^32^

### 3.9 Gene–drug interactions

#### 3.9.1 Efavirenz

Haas and Acosta characterised the effect of *CYP2B6* genotypes on the pharmacokinetic interaction between dolutegravir and efavirenz—a known inducer of hepatic drug‐metabolising enzymes. They found that the minimum plasma concentration of dolutegravir decreased by 60% in normal CYP2B6 metabolisers and by 85% in intermediate or slow metabolisers.^46^ Regarding weight gain, the *CYP2B6* slow efavirenz‐metaboliser genotype was not associated with greater weight gain 48 weeks after switching from efavirenz‐based to dolutegravir‐based cART, although weight gain has been reported with other INSTIs like elvitegravir and raltegravir.^47^, ^48^ Furthermore, Griesel et al. reported that weight gain was similar in CYP2B6 extensive metabolisers in both efavirenz and dolutegravir users and therefore suggested that impaired weight gain among CYP2B6 slow or intermediate metabolisers could explain the increased weight gain on dolutegravir compared with efavirenz.^49^

#### 3.9.2 Rifamycins

Covington et al. studied the effect of the *AADAC* rs1803155 genotype—which influences rifapentine metabolism—on the pharmacokinetic interaction between rifapentine and dolutegravir. *AADAC* rs1803155 was associated with lower dolutegravir trough concentrations at Day 28 in 30 HIV‐positive individuals receiving dolutegravir 50 mg twice daily alongside daily rifapentine/isoniazid. Thus, individuals with the slow metaboliser *AADAC* and normal metaboliser *UGT1A1* genotypes would have a higher risk of clinically significant drug–drug interactions between rifapentine/isoniazid and dolutegravir.^35^ Kengo et al. studied the interaction between rifampicin and *UGT1A1* on dolutegravir pharmacokinetics in a cohort of 44 Ugandan adult participants. Simulation of data from the study observed that the UGT1A1*1/*28, *1/*36 and *28/*28 genotypes were not associated with variation in metabolism of dolutegravir in individuals coinfected with HIV and tuberculosis who received both dolutegravir and standard or high‐dose rifampicin as compared with UGT1A1*1/*1.^50^

### 3.10 Gene–disease interactions

#### 3.10.1 Pre‐existing comorbidities

Carriers of the *SLC22A2* rs316019 C/A or A/A genotypes exhibited a higher prevalence of pre‐existing psychiatric comorbidities. A combination of pre‐existing psychiatric comorbidities and *SLC22A2* variants was a predictor of dolutegravir discontinuation due to neuropsychiatric symptoms. In individuals with pre‐existing psychiatric comorbidities, those carrying the *SLC22A2* C/C genotype were at the highest risk of dolutegravir discontinuation for any cause (34%, 26/76) and for neuropsychiatric symptoms (25%, 17/68). Furthermore, *SLC22A2* rs316019 was unevenly distributed among people living with HIV diagnosed with psychiatric disorders, suggesting a potential interaction between genetic factors and disease state.^39^

### 3.11 Other host‐specific factors

#### 3.11.1 Age, sex and body weight

Spector et al. reported a lower adjusted mean dolutegravir *C*_max_ difference in paediatric populations carrying ≥1 minor allele in *ABCG2* (rs2231142).^33^ However, studies in adults reported a higher dolutegravir *C*_max_ in individuals carrying the homozygous variant at the same locus in *ABCG2*.^32^, ^36^ There have been no studies that have investigated the interaction between genetic variation and the non‐genetic factors age, sex and body weight.

### 3.12 Methodological strengths and limitations

#### 3.12.1 Study design

The publications (peer‐reviewed and grey literature) included the following study designs: clinical trials only (10; phase I [1], phase II [2], phase III [5], phases I/II [1] and phases I/III [1]), observational cohort studies only (4), cross‐sectional studies only (6) and one combined both the observational cohort and clinical trial (phase III) designs (Table 1). Twelve of the peer‐reviewed articles shared partial or complete pharmacogenetic data from their primary studies (Table S1). One study did not control for multiple testing.^37^

#### 3.12.2 Sample size

Of 19 peer‐reviewed publications included, five had sample sizes <50, four from 51 to 100, seven from 101 to 500 and three from 501 to 1000 (Table 1). The two grey literature publications had sample sizes ranging from 101 to 500 and 501 to 1000, respectively (Table 2).

#### 3.12.3 Population coverage

Eight publications originated from North America (all from the United States); seven from sub‐Saharan Africa, including six from South Africa (five based on the ADVANCE trial) and one from Uganda; three from Europe (Italy and the United Kingdom); two from Asia (all from Japan); and one from South America (from Brazil) (Tables 1 and S1). The publications reported one or more ancestries, with 15 including populations of African ancestry, 12 of European ancestry and seven of Asian ancestry (Table 1).

#### 3.12.4 Genotyping approach

Most publications used targeted genotyping (18), followed by genome‐wide associated genotyping (8). Four of the publications that employed genome‐wide associated genotyping were from the ADVANCE trial. None of the publications reported using whole‐genome or whole‐exome sequencing (Table 1). A variety of genotyping technologies were documented (Table 3).

**TABLE 3:** Genotyping approaches used in the peer‐reviewed publications.

Author (year)	Scope	Technology	Method	Cost	Markers	Gene studied
Peer‐reviewed publications						
Chen S. (2014)	Targeted	PCR	PCR	(+)	1	*UGT1A1*, *CYP3A4*, *PXR*
	GWAS	Microarray	GEN‐PROBE AFFYMETRIX	(+++)	50k–2Mil	
Cindi Z. (2022)	GWAS	Microarray	Illumina Infinium Multi‐Ethnic Global BeadChip (MEGA^EX^)	(+++)	50k–2Mil	*ABCB1*, *ABCG2*, *NR1I2*, *UGT1A1*
Elliot E. R. (2020)	Targeted	PCR	TaqMan Genotyping Master mix	(+)	Up to 100	*ABCG2*, *UGT1A1*, *NR1I2*
		iPLEX	Agena MassARRAY iPLEX assay	(++)	40	
Griesel R. (2022)	GWAS	Microarray	Illumina Infinium Multi‐Ethnic Global BeadChip (MEGA^EX^)	(+++)	50k–2Mil	*UGT1A1*, *SLC22A2*
Borghetti A. (2019)	Targeted	Real‐time PCR	TaqMan SNP Genotyping Assays	(+)	1	*SLC22A2*
Cusato J. (2022)	Targeted	Real‐time PCR	CFX‐96 real‐time PCR	(+)	1	*SLC22A2*
Tsuchiya K. (2017)	Targeted	PCR	TAQMAN DRUG METABOLISM ASSAY	(+)	1	*UGT1A1*, *CYP3A4*, *ABCG2*
Mpofu R. (2024)	GWAS	Microarray	Illumina Infinium Multi‐Ethnic Global BeadChip (MEGA^EX^)	(+++)	50k–2Mil	*UGT1A1*, *SLC22A2*, *SLC47A1*, *SLC47A2*
Yagura H. (2017)	Targeted	PCR	PCR	(+)	1	*UGT1A1*
Spector S. A. (2024)	Targeted	PCR	rhAmp SNP GENOTYPING SYSTEM	(+)		*ABCG2*, *NR1I2*, *UGT1A1*
Cindi Z. (2021)	GWAS	Microarray	Illumina Infinium Multi‐Ethnic Global BeadChip (MEGA^EX^)	(+++)	50k–2Mil	*ABCG2*, *ABCC10*, *TMEM163*
Griesel R. (2021)	Targeted	iPLEX	MassARRAY GENOTYPING SYSTEM	(++)	40	*CYP2B6*
Leonard M. A. (2021)	Targeted	iPLEX	MassARRAY iPLEX Gold	(++)	40	*CYP2B6*, *UGT1A1*
	GWAS	Microarray	HumanCoreExome BeadChip assays (Illumina)	(++++)	25 000	
		Microarray	Illumina HumanHap 650Y array	(+++)	50k–2Mil	
		Microarray	Illumina 1M duo array	(+++)	50k–2Mil	
	Targeted	PCR	TaqMan	(+)	1	
Wu K. (2024)	Targeted	iPLEX	MassARRAY iPLEX Gold	(++)	40	*CYP2B6*, *UGT1A1*
	GWAS	Microarray	HumanCoreExome BeadChip assays (Illumina)	(++++)	25 000	
		Microarray	Illumina HumanHap 650Y array	(+++)	50k–2Mil	
		Microarray	Illumina 1M duo array	(+++)	50k–2Mil	
	Targeted	PCR	TaqMan	(+)	1	
Griesel R. (2024)	Targeted	iPLEX	MassARRAY genotyping system	(++)	40	*CYP2B6*
Murrell D. E. (2019)	Targeted	iPLEX	MassARRAY genotyping system	(++)	40	*CYP2D6*, *CYP2B6*, *SLCOB1*, *VKORC1*, *UGT1A1*
Kengo A. (2023)	Targeted	PCR	PCR	(+)	1	*UGT1A1*
Covington N. (2025)	Targeted	PCR	TaqMan	(+)	1	*UGT1A1*, *AADAC*, *NAT2*
Haas D. W. (2021)	Targeted	‐	‐	‐	‐	*CYP2B6*
De Greef J. (2025)	Targeted	PCR	TaqMan	(+)	1	*UGT1A1*, *SLC22A2*, *CYP3A4*, *CYP3A5*, *ABCB1*, *ABCG2*, *NR1I2*
Grey literature publications						
Ridolfi F. (2024)	Targeted	iPLEX	MassARRAY iPLEX Gold	(++)	40	*CYP2B6*, *UGT1A1*
Hulgan T. (2025)	‐	‐	‐	‐	‐	‐

#### 3.12.5 Phenotyping approach

The review showed a variety of phenotyping approaches, namely, pharmacokinetic data only (6), pharmacodynamic data only (10) and both pharmacokinetic and pharmacodynamic data (5) (Table 1).

## 4 DISCUSSION

This scoping review sought to synthesise current evidence on the pharmacogenomics of dolutegravir, focusing on pharmacokinetics, pharmacodynamics and the interplay between genetic and non‐genetic factors. We also appraised study design, sample size and genotyping and phenotyping strategies. Reduced‐function *UGT1A1* alleles (*6, *28, *37) are the most consistent determinants of dolutegravir exposure, with effects replicated in African, European and Asian populations, despite differences in allele frequencies between ancestries.^31^, ^32^, ^38^ Populations of African ancestry exhibit high genetic diversity, characterised by a high frequency of the reduced‐function alleles UGT1A1*28 and *37 alongside the rapid‐metaboliser variant UGT1A1*36.^31^, ^34^ In contrast, East Asian populations, particularly Japanese individuals, are uniquely influenced by the UGT1A1*6 variant, which has been linked to elevated dolutegravir *C*_trough_ and a higher incidence of neuropsychiatric adverse events.^38^ European‐descendent populations demonstrate a more homogenous profile, with a high prevalence of the wild‐type UGT1A1*1 allele, a common UGT1A1*28 reduced‐function allele and a near‐universal presence of the non‐expressor CYP3A5*3 variant.^31^, ^34^ Large‐scale genomic initiatives, such as the 1000 Genomes Project and All of Us Research Program, have further clarified these disparities by revealing that while common variants are shared globally, population‐specific alleles, particularly those within individuals of African ancestry, are often underrepresented in Eurocentric clinical trials.^51^, ^52^ Variations in ancestral distribution of genetic variants could explain potential differences in the efficacy and safety of dolutegravir across diverse global populations,^3^, ^31^, ^53^ but this needs further study.

*CYP3A4/CYP3A5* variants do not appear to be major determinants of dolutegravir exposure.^5^ Multiple pooled analyses and clinical pharmacokinetic studies report no consistent association between common *CYP3A4/CYP3A5* alleles (e.g., CYP3A4*22 and *CYP3A5* [*3/*6/*7]) and dolutegravir AUC/*C*_max_, implying that *UGT1A1* dominates dolutegravir clearance in vivo.^31^, ^32^, ^34^ However, current evidence is insufficient to warrant routine genotype‐guided dolutegravir dosing in the general population.^3^

The efflux transporter locus rs2231142 in the *ABCG2* gene is associated with higher dolutegravir *C*_max_ or *C*_trough_; however, the effect is inconsistent across populations.^32^, ^33^, ^34^ Tsuchiya et al. suggested that the low expression levels of efflux transporters in the intestines of individuals with the *ABCG2* rs2231142 variant could explain the higher plasma dolutegravir *C*_max_; however, the sample size of 42 Japanese adults is quite small, which could have increased the risk of false positives.^36^ In contrast to findings in adults, Spector et al. reported lower dolutegravir concentrations in children carrying the *ABCG2 rs2231142* variant. Dolutegravir AUC_24_ for genetic variants in *ABCG2* in children varies from −25% to +33%, which could explain some of the variability in dolutegravir pharmacokinetics.^33^ However, a larger African study of 284 adults did not find any associations between dolutegravir exposure and *ABCG2* which suggests the need for much larger studies in diverse populations.^34^ Data on age‐dependent expression of *ABCG2* also remain limited and more studies are needed.^54^

Genome‐wide analyses in African populations identified novel loci (*CAMKMT*, *MIR99AHG*) with potential effects on dolutegravir pharmacokinetics that did not reach definitive genome‐wide significance after correction. *CAMKMT* and *MIR99AHG* require in vitro and in vivo functional studies (e.g., expression QTLs, transporter and enzyme assays) to progress from statistical associations to causal inference.^34^ Furthermore, there is currently no clear mechanistic explanation for how *CAMKMT* or *MIR99AHG* variants might influence dolutegravir pharmacokinetics and therefore require replication and functional follow‐up.

Gene–gene and gene–drug pharmacokinetic interactions are of potential clinical importance but with limited replication. Combinations of *UGT1A1* poor‐metaboliser genotypes with *NR1I2* or *ABCG2* variants have been associated with additive or supra‐additive increases in dolutegravir exposure in pooled analyses.^32^ Similarly, concomitant use of inducers/inhibitors (notably rifamycins or efavirenz) modifies dolutegravir exposure in a genotype‐dependent manner.^35^, ^46^, ^47^, ^48^, ^49^ Gene–gene and gene–drug pharmacokinetic interaction signals are mechanistically credible and potentially clinically meaningful; however, most reports come from small datasets and require replication in adequately powered multi‐ancestry populations.^32^, ^33^, ^45^ Given the scarcity of literature on interactions between genetic variants and non‐genetic factors, it is important that future studies of dolutegravir disposition evaluate whether any relationship exists between genetic variation and host‐specific factors such as age, sex and body weight or body mass index (BMI). Regarding age, for instance, the literature shows disparate findings: The *ABCG2* rs2231142 variant is associated with higher dolutegravir *C*_max_ in adult populations^32^, ^36^ and a lower adjusted mean dolutegravir *C*_max_ in children carrying the same variant.^33^ On the other hand, body weight/BMI and sex are independent determinants of dolutegravir exposure.^32^, ^34^, ^42^ Polygenic and/or multivariable prediction models that integrate genotype, co‐medications, clinical characteristics and demographic covariates should be developed and validated, with evaluation of their incremental predictive value over clinical variables alone.

Pharmacogenetic associations with clinical outcomes (neuropsychiatric adverse events, weight gain, renal changes or viral suppression) showed a great deal of heterogeneity. Some evidence links *UGT1A1* and *SLC22A2* variants to neuropsychiatric adverse events, with suggestive gene–disease interactions in individuals with pre‐existing psychiatric comorbidities.^38^, ^39^, ^40^ Borghetti et al. suggested a potential *SLC22*A2‐mediated molecular pathophysiological mechanism for the occurrence of dolutegravir‐related neuropsychiatric effects; however, more prospective studies are needed to validate this relationship.^40^ Dolutegravir is also reported to downregulate mRNA and/or protein expression of the folate transporters *SLC19A1* and *SLC46A1* at the blood‐cerebrospinal fluid barrier and the blood–brain barrier in mice and human models.^55^ Folate is essential for brain health in all human age groups, being able to facilitate biochemical processes.^56^ Additionally, dolutegravir downregulates the mRNA expression of drug efflux transporters, including *ABCC1*, *ABCC4* and *ABCB1*, in mice, which could lead to dolutegravir accumulation in the brain.^55^ Whether these effects impact the mechanism of dolutegravir‐induced neuropsychiatric adverse effects is unclear and need further investigation in individuals living with HIV.

Weight gain with dolutegravir is inconsistently associated with genetic variation. Exploratory GWAS and candidate‐gene analyses have reported associations (*TMEM163* in one GWAS; *ABCG2* and *MC4R* loci in candidate‐gene studies), but none of these signals have been replicated across multiple independent datasets.^34^, ^43^ *TMEM163* encodes a zinc‐binding protein implicated in pancreatic β‐cell function.^57^, ^58^, ^59^, ^60^ Other *TMEM163* variants (rs6723108 and rs998451) have been linked to reduced fasting plasma insulin and HOMA‐IR, suggesting a role in glucose homeostasis.^61^ The relationship between *TMEM163* and dolutegravir‐related weight gain however requires replication. An alternative mechanism of weight gain is the potential role of *MC4R* variants. Melanocortins play an important role in appetite and weight regulation and may have a neuroendocrine contribution to INSTI‐related weight gain. However, the available evidence is still insufficient to support this conclusion.^43^ An in vitro study by MacMahon et al. observed that INSTIs can antagonise *MC4R*; however, this occurred at concentrations significantly higher than those expected in clinical settings, which is a less likely explanation for INSTI‐associated weight gain.^62^

Viral suppression has not been convincingly linked to common dolutegravir‐pharmacokinetic genotypes in the available studies. Small sample sizes in the studies by Spector et al. and Ridolfi et al. limit the ability to perform rigorous statistical comparisons between genetic variants in *ABCG2* or *UGT1A1* and viral suppression. In addition, the definitions of viral suppression differed between the two studies.^33^, ^45^ Spector et al. suggested that paediatric participants carrying polymorphisms in *UGT1A1* and *ABCG2* associated with increased dolutegravir concentrations potentially had a higher likelihood of viral suppression; however, this relationship was not statistically significant.^33^

Several limitations constrain confidence in, and generalisability of, the evidence from existing studies:

1. *Study design*: Nearly one third of publications were cross‐sectional. Yet, clinical trials and observational cohorts can provide a stronger level of evidence as they capture longitudinal changes in treatment response and adverse event occurrence relative to genotype. Furthermore, exploratory reports, such as that by Murrell et al.^37^ did not adequately correct for multiple testing, thereby increasing the likelihood of false‐positive results. Two publications pooled data across several phase I, phase II and phase III studies to increase the power of PGx analyses.^31^, ^32^ However, meta‐analyses require the identification and exclusion of individuals who have participated in more than one of the included clinical trials and those with inadequate pharmacokinetic and genetic data.^31^
2. *Sample size*: Most studies had small sample sizes (<500), which could lead to underpowered genetic analyses. It has been shown that pharmacogenetic phenotypes have larger effect sizes than those observed in complex traits and, thus, smaller sample sizes may be sufficient.^63^ However, this does not seem to be the case for variation in dolutegravir pharmacokinetics and pharmacodynamics as evidenced by the small sample sizes included in this review. Thus, the data reviewed here should be regarded as proof of concept and should be utilised to develop studies that are adequately powered. A sample size of at least 1000 participants has been suggested for meaningful pharmacogenomic analysis in clinical trials and at least 10 000 for genome‐wide association studies, even for common variants.^64^ Clearly, such sample sizes may not be possible or affordable in many cases, and feasibility should always be assessed before undertaking a study.
3. *Population coverage*: A third of included publications originated from South Africa and Uganda which are located in sub‐Saharan Africa, a region with the highest HIV burden and genetic diversity globally.^53^, ^65^ However, as most included publications from South Africa were primarily from the ADVANCE trial, there is limited representation of sub‐Saharan Africa's ethnic diversity, which constrains the generalisability of current findings.^34^, ^42^, ^44^, ^49^, ^66^ Increasing the number of publications from other settings within sub‐Saharan Africa will address the disparity. Most published datasets are concentrated in specific countries (the United States, South Africa, Japan and Italy), implying limited representation of individuals from other settings.^65^ Thus, more pharmacogenetic studies should be conducted in other regions to promote the global representation of genetic diversity.
4. *Genotyping approaches*: The genotyping strategies used across the reviewed publications varied from targeted genotyping of known genetic variants to genome‐wide association studies of broader sets of known genetic variants. However, none reported using whole‐genome or whole‐exome sequencing, possibly due to the higher cost of advanced methods coupled with limited expertise in certain settings, particularly in sub‐Saharan Africa, which restricts their widespread use. Targeted genotyping and GWAS are advantageous when hypothesis‐driven and cost‐effective hypothesis‐free approaches, respectively, together with cost‐efficiency are priorities. Furthermore, targeted genotyping is suited for small‐scale studies while GWAS is for broader analyses. GWAS can discover novel loci (recent African GWAS have reported novel signals) though require robust replication and functional follow‐up.^67^ The lack of uniformity of genotyping methods can make it difficult to compare results across studies, which limits the ability to draw firm conclusions.
5. *Phenotype heterogeneity and PK methods*: We note the lack of standardised phenotyping protocols in the included studies. Studies report diverse pharmacokinetic endpoints (*C*_max_, *C*_trough_, AUC, clearance and popPK‐derived parameters) collected with different sampling schemes. Clinically meaningful pharmacokinetic endpoints (e.g., AUC and *C*_trough_, which best reflect overall exposure and trough levels relevant to drug efficacy and toxicity) should be prioritised over heterogeneous measures like *C*_max_ alone. Clinical endpoints (neuropsychiatric AEs, discontinuation, weight gain and viral suppression) were also heterogeneously defined and often cross‐sectional or short term. For neuropsychiatric outcomes, the lack of standardised assessment tools (e.g., validated scales for anxiety, depression and sleep) likely contributed to the inconsistent findings; future consortia should prioritise consensus phenotype definitions.

### 4.1 Concluding statement

Reduced‐function *UGT1A1* alleles (*6, *28, *37) emerged as the most consistent determinants of increased dolutegravir exposure across African, European and Asian populations, reinforcing *UGT1A1* as the predominant genetic pathway for dolutegravir clearance. Genome‐wide association studies in African populations have identified novel candidate loci (e.g., *CAMKMT* and *MIR99AHG*) but these require replication. The current evidence base is also constrained by small sample sizes, heterogeneous study designs, overlapping datasets, non‐adjustment for multiple testing, limited ancestral diversity and inconsistent phenotyping. Therefore, priority should be given to well‐powered, multi‐ancestry, harmonised studies with functional validation to clarify the role of pharmacogenomics in tailoring dolutegravir‐based therapy.

## 5 FUTURE DIRECTIONS/RECOMMENDATIONS

1. Large, multi‐ancestry consortia with harmonised pharmacokinetic sampling and/or standardised clinical phenotyping, and pre‐specified analysis plans are essential. Meta‐analyses should prioritise individual participant data and consistent covariate adjustment (age, weight, sex, co‐medications and comorbidities). It is also important to understand how concomitant medications interact with dolutegravir and either increase or decrease the effect of a genetic variant.
2. Future well‐powered studies should replicate both leading and novel GWAS‐identified variants—beyond *UGT1A1* and *ABCG2*—in independent cohorts, assess their functional relevance and integrate these findings into the development of polygenic risk scores across globally diverse populations.
3. Investigate the pharmacogenetic basis of emerging adverse events, for example, hyperglycaemia, type II diabetes mellitus, sexual dysfunction and cardiovascular disease.
4. Conduct more pharmacogenomic studies in vulnerable populations, for example, paediatrics, pregnant women and the elderly, and relate to PK measures and clinical endpoints.
5. Standardising phenotype protocols, reporting and multiple‐testing correction through preregistration of genetic analyses in trials, consistent PK endpoints and rigorous multiplicity control should be inculcated in pharmacogenetic association studies.
6. Future research could prioritise mechanistic inquiry into the molecular and physiological pathways underlying dolutegravir‐associated adverse drug reactions (including neuropsychiatric, metabolic, renal and emerging toxicities), ahead of large‐scale longitudinal studies of genotype‐guided interventions, which are resource intensive.
7. Depending on the result of genome‐wide association studies, whole‐genome sequencing should be undertaken to identify novel variants (particularly rare variants) associated with dolutegravir phenotypes. The availability of GWAS and sequencing data may enable the development of polygenic risk scores related to dolutegravir exposure, efficacy and safety, but this will need careful replication.
8. Integrate pharmacogenomic data with other omics data, for example, transcriptomics, proteomics and metabolomics, to provide comprehensive understanding of dolutegravir efficacy and toxicity.
9. Integrating therapeutic drug monitoring with genotype‐guided dosing provides a more translatable framework than genotyping alone, by addressing both metabolic inheritance and environmental factors. However, its scaling requires investment in diagnostic infrastructure to realise its full clinical potential.

### 5.1 Nomenclature of Targets and Ligands

Key protein targets and ligands in this article are hyperlinked to corresponding entries in http://www.guidetopharmacology.org and are permanently archived in the Concise Guide to PHARMACOLOGY 2021/22.^68^, ^69^, ^70^, ^71^

## AUTHOR CONTRIBUTIONS

All authors contributed equally to the manuscript.

## CONFLICT OF INTEREST STATEMENT

M.P. currently receives partnership funding, paid to the University of Liverpool, for the following: MRC Medicines Development Fellowship Scheme (co‐funded by MRC, GSK and AZ). He has developed an HLA genotyping panel with MC Diagnostics but does not benefit financially from this. He is part of the IMI Consortium ARDAT (www.ardat.org); none of these funding sources have been used for the current research.

## Supporting information


**Data S1.** Search strings for peer‐reviewed publications.

**Data S2.** Data extraction tool for peer‐reviewed publications.

**Table S1:** Grouping of publications based on shared primary datasets.

## Data Availability

Data sharing is not applicable to this article as no datasets were generated or analysed during the current study.

## References

[1] World Health Organization . Transition to new antiretroviral drugs in HIV programmes: clinical and programmatic considerations. 2017. https://iris.who.int/bitstream/handle/10665/255887/WHO‐HIV‐2017.23‐eng.pdf;jsessionid=D77E5942645D0E27CE931E9B62262CAF?sequence=1

[2] Min S , Song I , Borland J , et al. Pharmacokinetics and safety of S/GSK1349572, a next‐generation HIV integrase inhibitor, in healthy volunteers. Antimicrob Agents Chemother. 2010;54(1):254‐258. doi:10.1128/AAC.00842-09 PMC2798521 19884365

[3] Bevers LAH , Jensen RL , Owen A , Colbers A , Carr DF , Burger DM . Genetic variation on dolutegravir pharmacokinetics and relation to safety and efficacy outcomes: a systematic review. Pharmacogenomics. 2024;25(14–15):623‐635. doi:10.1080/14622416.2024.2441104 PMC11703464 39697075

[4] Roden DM , Wilke RA , Kroemer HK , Stein CM . Pharmacogenomics: the genetics of variable drug responses. Circulation. 2011;123(15):1661‐1670. doi:10.1161/CIRCULATIONAHA.109.914820 PMC3093198 21502584

[5] Reese MJ , Savina PM , Generaux GT , et al. In vitro investigations into the roles of drug transporters and metabolizing enzymes in the disposition and drug interactions of dolutegravir, a HIV integrase inhibitor. Drug Metab Dispos. 2013;41(2):353‐361. doi:10.1124/dmd.112.048918 23132334

[6] Liu SN , Lu JBL , Watson CJW , Lazarus P , Desta Z , Gufford BT . Mechanistic assessment of extrahepatic contributions to glucuronidation of integrase strand transfer inhibitors. Drug Metab Dispos. 2019;47(5):535‐544. doi:10.1124/dmd.118.085035 PMC6474910 30804050

[7] Gammal RS , Court MH , Haidar CE , et al. Clinical Pharmacogenetics Implementation Consortium (CPIC) guideline for UGT1A1 and atazanavir prescribing. Clin Pharmacol Ther. 2016;99(4):363‐369. doi:10.1002/cpt.269 PMC4785051 26417955

[8] Teh LK , Hashim H , Zakaria ZA , Salleh MZ . Polymorphisms of UGT1A1*6, UGT1A1*27 & UGT1A1*28 in three major ethnic groups from Malaysia. Indian J Med Res. 2012;136(2):249‐259. PMC3461737 22960892

[9] Pasternak AL , Crews KR , Caudle KE , et al. The impact of the UGT1A1*60 allele on bilirubin serum concentrations. Pharmacogenomics. 2017;18(1):5‐16. doi:10.2217/pgs-2016-0135 27967321

[10] Castellino S , Moss L , Wagner D , et al. Metabolism, excretion, and mass balance of the HIV‐1 integrase inhibitor dolutegravir in humans. Antimicrob Agents Chemother. 2013;57(8):3536‐3546. doi:10.1128/AAC.00292-13 PMC3719771 23669385

[11] Ding X , Kaminsky LS . Human extrahepatic cytochromes P450: function in xenobiotic metabolism and tissue‐selective chemical toxicity in the respiratory and gastrointestinal tracts. Annu Rev Pharmacol Toxicol. 2003;43(1):149‐173. doi:10.1146/annurev.pharmtox.43.100901.140251 12171978

[12] Hukkanen J , Pelkonen O , Hakkola J , Raunio H . Expression and regulation of xenobiotic‐metabolizing cytochrome P450 (CYP) enzymes in human lung. Crit Rev Toxicol. 2002;32(5):391‐411. doi:10.1080/20024091064273 12389869

[13] Shimada T , Hayes CL , Yamazaki H , et al. Activation of chemically diverse procarcinogens by human cytochrome P‐450 1B1. Cancer Res. 1996;56(13):2979‐2984. 8674051

[14] Wang X , Cerrone M , Ferretti F , et al. Pharmacokinetics of dolutegravir 100 mg once daily with rifampicin. Int J Antimicrob Agents. 2019;54(2):202‐206. doi:10.1016/j.ijantimicag.2019.04.009 31002950

[15] Zhang B , Xie W , Krasowski MD . PXR: a xenobiotic receptor of diverse function implicated in pharmacogenetics. Pharmacogenomics. 2008;9(11):1695‐1709. doi:10.2217/14622416.9.11.1695 PMC2593625 19018724

[16] de Boer MG , van den Berk GE , van Holten N , et al. Intolerance of dolutegravir‐containing combination antiretroviral therapy regimens in real‐life clinical practice. Aids. 2016;30(18):2831‐2834. 10.1097/QAD.0000000000001279 27824625

[17] Elzi L , Erb S , Furrer H , et al. Adverse events of raltegravir and dolutegravir. Aids. 2017;31(13):1853‐1858. doi:10.1097/QAD.0000000000001590 PMC5566167 28692533

[18] Hoffmann C , Welz T , Sabranski M , et al. Higher rates of neuropsychiatric adverse events leading to dolutegravir discontinuation in women and older patients. HIV Med. 2017;18(1):56‐63. doi:10.1111/hiv.12468 27860104

[19] Clotet B , Feinberg J , van Lunzen J , et al. Once‐daily dolutegravir versus darunavir plus ritonavir in antiretroviral‐naive adults with HIV‐1 infection (FLAMINGO): 48 week results from the randomised open‐label phase 3b study. Lancet. 2014;383(9936):2222‐2231. doi:10.1016/S0140-6736(14)60084-2 24698485

[20] Hoffmann C , Llibre JM . Neuropsychiatric adverse events with dolutegravir and other integrase strand transfer inhibitors. AIDS Rev. 2019;21(1):4‐10. doi:10.24875/AIDSRev.19000023 30899113

[21] Orrell C , Hagins DP , Belonosova E , et al. Fixed‐dose combination dolutegravir, abacavir, and lamivudine versus ritonavir‐boosted atazanavir plus tenofovir disoproxil fumarate and emtricitabine in previously untreated women with HIV‐1 infection (ARIA): week 48 results from a randomised, open‐label, non‐inferiority, phase 3b study. Lancet HIV. 2017;4(12):e536‐e546. doi:10.1016/S2352-3018(17)30095-4 28729158

[22] Raffi F , Rachlis A , Stellbrink HJ , et al. Once‐daily dolutegravir versus raltegravir in antiretroviral‐naive adults with HIV‐1 infection: 48 week results from the randomised, double‐blind, non‐inferiority SPRING‐2 study. Lancet. 2013;381(9868):735‐743. doi:10.1016/S0140-6736(12)61853-4 23306000

[23] van Lunzen J , Maggiolo F , Arribas JR , et al. Once daily dolutegravir (S/GSK1349572) in combination therapy in antiretroviral‐naive adults with HIV: planned interim 48 week results from SPRING‐1, a dose‐ranging, randomised, phase 2b trial. Lancet Infect Dis. 2012;12(2):111‐118. doi:10.1016/S1473-3099(11)70290-0 22018760

[24] Walmsley SL , Antela A , Clumeck N , et al. Dolutegravir plus abacavir‐lamivudine for the treatment of HIV‐1 infection. N Engl J Med. 2013;369(19):1807‐1818. doi:10.1056/NEJMoa1215541 24195548

[25] Peters MD , Godfrey CM , Khalil H , McInerney P , Parker D , Soares CB . Guidance for conducting systematic scoping reviews. Int J Evid Based Healthc. 2015;13(3):141‐146. doi:10.1097/XEB.0000000000000050 26134548

[26] Tricco AC , Lillie E , Zarin W , et al. PRISMA extension for scoping reviews (PRISMA‐ScR): checklist and explanation. Ann Intern Med. 2018;169(7):467‐473. doi:10.7326/M18-0850 30178033

[27] ClinPGx . ClinPGx. https://www.clinpgx.org/. Accessed 18 August 2025.

[28] ProQuest . ProQuest Dissertations & Theses Global. https://about.proquest.com/en/products-services/pqdtglobal/. Accessed 18 August 2025.

[29] US National Library of Medicine . ClinicalTrials.Gov. https://clinicaltrials.gov/. Accessed 18 August 2025.

[30] World Health Organization . Institutional repository for information sharing. https://iris.who.int/. Accessed 18 August 2025.

[31] Chen S , St Jean P , Borland J , et al. Evaluation of the effect of UGT1A1 polymorphisms on dolutegravir pharmacokinetics. Pharmacogenomics. 2014;15(1):9‐16. doi:10.2217/pgs.13.190 24329186

[32] Elliot ER , Neary M , Else L , et al. Genetic influence of ABCG2, UGT1A1 and NR1I2 on dolutegravir plasma pharmacokinetics. J Antimicrob Chemother. 2020;75(5):1259‐1266. doi:10.1093/jac/dkz558 32011683

[33] Spector SA , Brummel SS , Chang A , Wiznia A , Ruel TD , Acosta EP . Impact of genetic variants in ABCG2, NR1I2, and UGT1A1 on the pharmacokinetics of dolutegravir in children. J Acquir Immune Defic Syndr (1999). 2024;95(3):297‐303. 10.1097/QAI.0000000000003358 PMC10922521 38180896

[34] Cindi Z , Kawuma AN , Maartens G , et al. Pharmacogenetics of dolutegravir plasma exposure among southern Africans with human immunodeficiency virus. J Infect Dis. 2022;226(9):1616‐1625. doi:10.1093/infdis/jiac174 PMC9624457 35512135

[35] Covington N , Luetkemeyer AF , Imperial MZ , et al. Pharmacogenetics of plasma dolutegravir exposure during 1‐month rifapentine/isoniazid treatment of latent tuberculosis. Pharmacogenet Genomics. 2025;35(4):140‐144. doi:10.1097/FPC.0000000000000562 PMC12043259 39960813

[36] Tsuchiya K , Hayashida T , Hamada A , Oki S , Oka S , Gatanaga H . High plasma concentrations of dolutegravir in patients with ABCG2 genetic variants. Pharmacogenet Genomics. 2017;27(11):416‐419. doi:10.1097/FPC.0000000000000308 28858994

[37] Murrell DE , Cluck DB , Moorman JP , et al. HIV integrase inhibitor pharmacogenetics: an exploratory study. Clin Drug Investig. 2019;39(3):285‐299. doi:10.1007/s40261-018-0739-9 30623371

[38] Yagura H , Watanabe D , Kushida H , et al. Impact of UGT1A1 gene polymorphisms on plasma dolutegravir trough concentrations and neuropsychiatric adverse events in Japanese individuals infected with HIV‐1. BMC Infect Dis. 2017;17(1):622. doi:10.1186/s12879-017-2717-x PMC5603066 28915895

[39] Cusato J , Borghetti A , Teti E , et al. Dolutegravir discontinuation for neuropsychiatric symptoms in people living with HIV and their outcomes after treatment change: a pharmacogenetic study. Metabolites. 2022;12(12):1202. doi:10.3390/metabo12121202 PMC9781993 36557240

[40] Borghetti A , Calcagno A , Lombardi F , et al. SLC22A2 variants and dolutegravir levels correlate with psychiatric symptoms in persons with HIV. J Antimicrob Chemother. 2019;74(4):1035‐1043. doi:10.1093/jac/dky508 30561642

[41] De Greef J , Yombi JC , Vincent A , et al. Dolutegravir and risk of neuropsychiatric adverse events: a pharmacogenetic study. J Infect Dis. 2025;231(6):1425‐1429. doi:10.1093/infdis/jiaf098 40036182

[42] Griesel R , Sinxadi P , Kawuma A , et al. Pharmacokinetic and pharmacogenetic associations with dolutegravir neuropsychiatric adverse events in an African population. J Antimicrob Chemother. 2022;77(11):3110‐3117. doi:10.1093/jac/dkac290 PMC7613765 36031789

[43] Hulgan T , Erlandson KM , Bradford Y , et al. Melanocortin‐4 receptor gene variants and weight change following switch to integrase inhibitor‐containing antiretroviral therapy. Open Forum Infect Dis. 2025;12(Suppl 1):ofaf696. 10.1093/ofid/ofaf696 PMC12662048 41322244

[44] Mpofu R , Kawuma AN , Wasmann RE , et al. Determinants of early change in serum creatinine after initiation of dolutegravir‐based antiretroviral therapy in South Africa. Br J Clin Pharmacol. 2024;90(5):1247‐1257. doi:10.1111/bcp.16009 PMC12573106 38332460

[45] Ridolfi F , Amorim G , Haas DW , et al. Pharmacogenomic associations with HIV‐1 virologic suppression in TB/HIV patients. Res Sq. 2024.

[46] Haas DW , Acosta EP . Implications of Efavirenz pharmacogenetics when switching from efavirenz‐ to dolutegravir‐containing antiretroviral regimens. Clin Infect Dis. 2021;72(10):1820‐1822. doi:10.1093/cid/ciaa975 PMC8130017 32667979

[47] Leonard MA , Cindi Z , Bradford Y , et al. Efavirenz pharmacogenetics and weight gain following switch to integrase inhibitor‐containing regimens. Clin Infect Dis. 2021;73(7):e2153‐e2163. doi:10.1093/cid/ciaa1219 PMC8492125 32829410

[48] Wu K , Koethe J , Hulgan T , et al. Pharmacogenetics of weight gain following switch from efavirenz‐ to integrase inhibitor‐containing regimens. Pharmacogenet Genomics. 2024;34(2):25‐32. doi:10.1097/FPC.0000000000000515 PMC10732300 37910437

[49] Griesel R , Maartens G , Chirehwa M , et al. CYP2B6 genotype and weight gain differences between dolutegravir and efavirenz. Clin Infect Dis. 2021;73(11):e3902‐e3909. doi:10.1093/cid/ciaa1073 PMC8653639 32960272

[50] Kengo A , Nabisere R , Gausi K , et al. Dolutegravir pharmacokinetics in Ugandan patients with TB and HIV receiving standard‐ versus high‐dose rifampicin. Antimicrob Agents Chemother. 2023;67(11):e0043023. doi:10.1128/aac.00430-23 PMC10648962 37850738

[51] The All of Us Research Program Genomics Investigators . Genomic data in the all of us research program. Nature. 2024;627(8003):340‐346. doi:10.1038/s41586-023-06957-x PMC10937371 38374255

[52] Auton A , Brooks LD , Durbin RM , et al. A global reference for human genetic variation. Nature. 7571;526(7571):68‐74. doi:10.1038/nature15393 PMC4750478 26432245

[53] Tishkoff SA , Reed FA , Friedlaender FR , et al. The genetic structure and history of Africans and African Americans. Science. 2009;324(5930):1035‐1044. doi:10.1126/science.1172257 PMC2947357 19407144

[54] Felmlee MA , Zhang Q . Ontogeny of drug transporters. Drug Transporters. 2022;393‐412. doi:10.1002/9781119739883.ch19

[55] Huang C , Hoque MT , Qu QR , Henderson J , Bendayan R . Antiretroviral drug dolutegravir induces inflammation at the mouse brain barriers. FASEB J. 2024;38(13):e23790. doi:10.1096/fj.202400558R PMC13688087 38982638

[56] McGarel C , Pentieva K , Strain JJ , McNulty H . Emerging roles for folate and related B‐vitamins in brain health across the lifecycle. Proc Nutr Soc. 2015;74(1):46‐55. doi:10.1017/S0029665114001554 25371067

[57] Cuajungco MP , Basilio LC , Silva J , et al. Cellular zinc levels are modulated by TRPML1‐TMEM163 interaction. Traffic. 2014;15(11):1247‐1265. doi:10.1111/tra.12205 PMC4205267 25130899

[58] Lemaire K , Chimienti F , Schuit F . Zinc transporters and their role in the pancreatic β‐cell. J Diabetes Investig. 2012;3(3):202‐211. doi:10.1111/j.2040-1124.2012.00199.x PMC4014939 24843567

[59] Nicolson TJ , Bellomo EA , Wijesekara N , et al. Insulin storage and glucose homeostasis in mice null for the granule zinc transporter ZnT8 and studies of the type 2 diabetes‐associated variants. Diabetes. 2009;58(9):2070‐2083. doi:10.2337/db09-0551 PMC2731533 19542200

[60] Tabassum R , Chauhan G , Dwivedi OP , et al. Genome‐wide association study for type 2 diabetes in Indians identifies a new susceptibility locus at 2q21. Diabetes. 2013;62(3):977‐986. doi:10.2337/db12-0406 PMC3581193 23209189

[61] Pisanu C , Williams MJ , Ciuculete DM , et al. Evidence that genes involved in hedgehog signaling are associated with both bipolar disorder and high BMI. Transl Psychiatry. 2019;9(1):315. doi:10.1038/s41398-019-0652-x PMC6872724 31754094

[62] McMahon C , Trevaskis JL , Carter C , et al. Lack of an association between clinical INSTI‐related body weight gain and direct interference with MC4 receptor (MC4R), a key central regulator of body weight. PLoS ONE. 2020;15(2):e0229617. doi:10.1371/journal.pone.0229617 PMC7048285 32109250

[63] Maranville JC , Cox NJ . Pharmacogenomic variants have larger effect sizes than genetic variants associated with other dichotomous complex traits. Pharmacogenomics J. 2016;16(4):388‐392. doi:10.1038/tpj.2015.47 PMC4704992 26149738

[64] Bailey KR , Cheng C . Conference scene: the great debate: genome‐wide association studies in pharmacogenetics research, good or bad? Pharmacogenomics. 2010;11(3):305‐308. doi:10.2217/pgs.10.6 PMC5012174 20235786

[65] UNAIDS . 2024 global AIDS report—the urgency of now: AIDS at a crossroads. 2024. https://www.unaids.org/en/resources/documents/2024/global-aids-update-2024

[66] Cindi Z , Maartens G , Bradford Y , et al. Genetic associations with weight gain among South Africans who initiated dolutegravir‐containing and tenofovir‐containing regimens. J Acquir Immune Defic Syndr (1999). 2021;87(3):1002‐1009. 10.1097/QAI.0000000000002661 PMC8192425 33625064

[67] Kockum I , Huang J , Stridh P . Overview of genotyping technologies and methods. Curr Protoc. 2023;3(4):e727. doi:10.1002/cpz1.727 PMC13528262 37026777

[68] Alexander SP , Christopoulos A , Davenport AP , et al. The CONCISE GUIDE TO PHARMACOLOGY 2021/22: G protein‐coupled receptors. Br J Pharmacol. 2021;178(Suppl 1):S27‐S156. doi:10.1111/bph.15538 34529832

[69] Alexander SP , Cidlowski JA , Kelly E , et al. The CONCISE GUIDE TO PHARMACOLOGY 2021/22: nuclear hormone receptors. Br J Pharmacol. 2021;178(Suppl 1):S246‐S263. doi:10.1111/bph.15540 PMC9513947 34529827

[70] Alexander SP , Fabbro D , Kelly E , et al. The CONCISE GUIDE TO PHARMACOLOGY 2021/22: enzymes. Br J Pharmacol. 2021;178(Suppl 1):S313‐S411. doi:10.1111/bph.15542 34529828

[71] Alexander SP , Kelly E , Mathie A , et al. The CONCISE GUIDE TO PHARMACOLOGY 2021/22: transporters. Br J Pharmacol. 2021;178(Suppl 1):S412‐S513. doi:10.1111/bph.15543 34529826

