# Supplementary material for: Pharmacogenomics of dolutegravir: A scoping review of evidence, gaps and clinical implications
Source: Br J Clin Pharmacol. 2026 Jul 28;92(11):3716–32. doi: 10.1002/bcp.70721 (PMC13714618; doi:10.1002/bcp.70721)
Supplement: Supplementary file 1 [file BCP-92-3716-s001.docx]

| **Supplementary material II: Data extraction tool for peer-reviewed publications** | | | | | | | | | | | | | | | |
| --- | --- | --- | --- | --- | --- | --- | --- | --- | --- | --- | --- | --- | --- | --- | --- |
| **First Author (Year)** | **Journal** | **Title** | **Objectives** | **Study design** | **Sample size** | **Country** | **Phenotyping Approach** | | **Genotyping Approach** | | **Ancestry** | | | |  |
|  |  |  |  |  |  |  | **PK** | **PD** | **Targeted** | **GWAS** | **African** | **European** | **Asian** | **Unknown/Others** |  |
|  |  |  |  |  |  |  |  |  |  |  |  |  |  |  |  |
