# Supplementary material for: Pharmacogenomics of dolutegravir: A scoping review of evidence, gaps and clinical implications
Source: Br J Clin Pharmacol. 2026 Jul 28;92(11):3716–32. doi: 10.1002/bcp.70721 (PMC13714618; doi:10.1002/bcp.70721)
Supplement: Supplementary file 2 [file BCP-92-3716-s002.docx]

**Supplementary material I: Search Strings for Peer-reviewed publications**

1. ((((dolutegravir) OR (tivicay)) OR (GSK1349572)) OR (S/GSK1349572))
2. (((((((pharmacogenetic*) OR (pharmacogenomic*)) OR (genetic*)) OR (genomic*)) OR (gene*)) OR ("single nucleotide polymorphism") OR (SNP)))
3. (((((((((pharmacokinetic*) OR (PK)) OR (absorption)) OR (distribution)) OR (metabolism)) OR (elimination)) OR ("drug clearance") OR (clearance)) OR (enzyme*)) OR (transporter*))
4. ((((((((((((((((((pharmacodynamic*) OR (PD)) OR ("drug response")) OR ("treatment response")) OR ("treatment failure")) OR ("virologic suppression")) OR ("virological suppression")) OR ("viral suppression")) OR ("virologic failure")) OR ("virological failure")) OR ("viral failure")) OR (efficacy)) OR ("adverse event*")) OR (symptom*)) OR ("adverse effect*")) OR ("adverse drug reaction*")) OR (ADR) OR (ADRs) OR ("drug effect*") OR (toxicity) OR (intolerance)))
5. #1 AND #2
6. #3 OR #4
7. #5 AND #6
