# Supplementary material for: Pharmacogenomics of dolutegravir: A scoping review of evidence, gaps and clinical implications
Source: Br J Clin Pharmacol. 2026 Jul 28;92(11):3716–32. doi: 10.1002/bcp.70721 (PMC13714618; doi:10.1002/bcp.70721)
Supplement: Supplementary file 3 [file BCP-92-3716-s003.docx]

| **Table S1: Grouping of publications based on shared primary datasets** | | | |
| --- | --- | --- | --- |
| **First Author (year)** | **Journal** | **Title** | **Primary Trial/Source of Data** |
| **Shared primary datasets** | |  |  |
| Cindi Z  (2022) | J Infect Dis | Pharmacogenetics of Dolutegravir plasma exposure among Southern Africans with HIV | ADVANCE Trial in South Africa |
| Griesel R  (2022) | J Antimicrob Chemother | Pharmacokinetic and pharmacogenetic associations with dolutegravir neuropsychiatric adverse events in Africa | ADVANCE Trial in South Africa |
| Mpofu R  (2024) | Br J Clin Pharmacol | Determinants of early change in serum creatinine after initiation of dolutegravir-based ART in South Africa | ADVANCE Trial in South Africa |
| Cindi Z  (2021) | J Acquir Immune Defic Syndr | Genetic Associations with Weight Gain among South Africans on Dolutegravir and Tenofovir regimens | ADVANCE Trial in South Africa |
| Griesel R  (2021) | Clin Infect Dis | CYP2B6 Genotype and Weight Gain Differences Between Dolutegravir and Efavirenz | ADVANCE Trial in South Africa |
| Leonard MA  (2021) | Clin Infect Dis | Efavirenz Pharmacogenetics and Weight Gain Following Switch to Integrase Inhibitor Regimens | ACTG A5095, A5142, A5202 in USA |
| Wu K  (2024) | Pharmacogenet Genomics | Pharmacogenetics of weight gain following switch from Efavirenz to Integrase inhibitors | ACTG A5001, A5322 in USA |
| Elliot ER  (2020) | J Antimicrob Chemother | Genetic influence of ABCG2, UGT1A1, NR1I2 on Dolutegravir plasma pharmacogenetics | ACTG A5359, A5322, A5321, A5384 (NCT02219217, NCT02509195, NCT03094507, NCT02351908) in UK |
| Haas DW  (2021) | Clin Infect Dis | Implications of Efavirenz pharmacogenetics when switching from Efavirenz to Dolutegravir-containing regimens | ING114005 in USA |
| Chen S  (2014) | Pharmacogenomics | Evaluation of the effect of UGT1A1 polymorphisms on dolutegravir pharmacokinetics | ING111603, ING111604, ING112934, ING113068, ING113096, ING114005, ING114819, ING113099, ING111521 in USA |
| Borghetti A  (2019) | J Antimicrob Chemother | SLC22A2 variants and dolutegravir levels correlate with psychiatric symptoms in persons with HIV | Observational study at two clinical centres in Italy |
| Cusato J  (2022) | Metabolites | Dolutegravir discontinuation for neuropsychiatric symptoms in people living with HIV | Observational study at three clinical centres in Italy |
| **Distinct primary studies** | |  |  |
| Griesel R  (2024) | J Acquir Immune Defic Syndr | Pharmacokinetics of Single vs. Double-Dose Dolutegravir after switching from Efavirenz-based regimen | ARTIST Trial in South Africa |
| Yagura H  (2017) | BMC Infect Dis | Impact of UGT1A1 polymorphisms on dolutegravir levels and neuropsychiatric events in Japanese individuals | Observational study in Japan |
| Spector SA  (2024) | J Acquir Immune Defic Syndr | Impact of Genetic Variants in ABCG2, NR1I2, and UGT1A1 on Dolutegravir Pharmacokinetics in Children | IMPAACT P1093 Trial in USA |
| Kengo A  (2023) | Antimicrob Agents Chemother | Dolutegravir pharmacokinetics in Ugandan patients with TB on standard vs. high-dose rifampicin | SAEFRIF study in Uganda |
| Tsuchiya K  (2017) | Pharmacogenet Genomics. | High plasma concentrations of dolutegravir in patients with ABCG2 genetic variants | Observational study in Japan |
| Covington N  (2025) | Pharmacogenet Genomics | Pharmacogenetics of plasma dolutegravir exposure during 1-month rifapentine/isoniazid treatment of latent tuberculosis | Observational study in USA |
| Murrell DE  (2019) | Clin Drug Investig. | HIV Integrase Inhibitor Pharmacogenetics: An Exploratory Study | Observational study in USA |
| De Greef  (2025) | J Infect Dis. | Dolutegravir and Risk of Neuropsychiatric Adverse Events: A Pharmacogenetic Study | Observational study in Belgium |
